# Vitamin D Deficiency, Excessive Gestational Weight Gain, and Oxidative Stress Predict Small for Gestational Age Newborns Using an Artificial Neural Network Model

**DOI:** 10.3390/antiox11030574

**Published:** 2022-03-17

**Authors:** Otilia Perichart-Perera, Valeria Avila-Sosa, Juan Mario Solis-Paredes, Araceli Montoya-Estrada, Enrique Reyes-Muñoz, Ameyalli M. Rodríguez-Cano, Carla P. González-Leyva, Maribel Sánchez-Martínez, Guadalupe Estrada-Gutierrez, Claudine Irles

**Affiliations:** 1 Nutrition and Bioprogramming Coordination, Instituto Nacional de Perinatologia, Mexico City 11000, Mexico; otiliaperichart@inper.gob.mx (O.P.-P.); rocameyalli@gmail.com (A.M.R.-C.); syh5ac@virginia.edu (C.P.G.-L.); 2 Department of Physiology and Cellular Development, Instituto Nacional de Perinatologia, Mexico City 11000, Mexico; valfer2701@gmail.com; 3 Department of Human Genetics and Genomics, Instituto Nacional de Perinatologia, Mexico City 11000, Mexico; juan.mario.sp@gmail.com; 4 Coordination of Gynecological and Perinatal Endocrinology, Instituto Nacional de Perinatologia, Mexico City 11000, Mexico; ara_mones@hotmail.com (A.M.-E.); dr.enriquereyes@gmail.com (E.R.-M.); 5 Department of Immunobiochemistry, Instituto Nacional de Perinatologia, Mexico City 11000, Mexico; maribel71sm@yahoo.com.mx; 6 Research Direction, Instituto Nacional de Perinatologia, Mexico City 11000, Mexico; gpestrad@gmail.com

**Keywords:** pregnancy, neural network, oxidative damage, neonate, small for gestational age

## Abstract

(1) Background: Size at birth is an important early determinant of health later in life. The prevalence of small for gestational age (SGA) newborns is high worldwide and may be associated with maternal nutritional and metabolic factors. Thus, estimation of fetal growth is warranted. (2) Methods: In this work, we developed an artificial neural network (ANN) model based on first-trimester maternal body fat composition, biochemical and oxidative stress biomarkers, and gestational weight gain (GWG) to predict an SGA newborn in pregnancies with or without obesity. A sensibility analysis to classify maternal features was conducted, and a simulator based on the ANN algorithm was constructed to predict the SGA outcome. Several predictions were performed by varying the most critical maternal features attained by the model to obtain different scenarios leading to SGA. (3) Results: The ANN model showed good performance between the actual and simulated data (R^2^ = 0.938) and an AUROC of 0.8 on an independent dataset. The top-five maternal predictors in the first trimester were protein and lipid oxidation biomarkers (carbonylated proteins and malondialdehyde), GWG, vitamin D, and total antioxidant capacity. Finally, excessive GWG and redox imbalance predicted SGA newborns in the implemented simulator. Significantly, vitamin D deficiency also predicted simulated SGA independently of GWG or redox status. (4) Conclusions: The study provided a computational model for the early prediction of SGA, in addition to a promising simulator that facilitates hypothesis-driven constructions, to be further validated as an application.

## 1. Introduction

Size at birth is an indicator of metabolic and nutrition programming of diseases [1,2]. Small for gestational age (SGA) is a commonly accepted proxy measure of IUGR (Intrauterine growth restriction) and has been defined as a birth weight less than the 10th percentile for a specific completed gestational age and sex of a reference population [3,4]. Infants born SGA carry a considerably higher risk of mortality and morbidity in the neonatal period and beyond, with a higher risk of neonatal infections, jaundice, polycythemia, hypoglycemia, poor feeding, and hypothermia [4]; as well as an increased risk of delayed neurodevelopment and poor linear growth [5]. In addition, low birth weight and SGA increase the risk of developing obesity, diabetes, and cardiovascular diseases in adulthood [6]. While nutritional deficiency is expected to be the most significant contributor to SGA in low and middle-income countries, other causal mechanisms, such as maternal infections, placental insufficiency, pregnancy morbidity, and environmental exposures, contribute in these settings [4].

The prevalence of SGA newborns is high around the world. In 2012, approximately 19.3% of live births (range 17.6 to 31.9%) were born SGA in low and middle-income countries [4]. In 2010, in the Latin America and Caribbean region, the prevalence was 11% in term infants and 2% in preterm infants [3].

Fetal growth is a complex phenomenon resulting from multiple intrauterine environmental factors, where nutrient and oxygen availability are essential, together with hormonal, adipokine, oxidative stress (OS), and inflammation status [7,8]. In women with obesity, an increase in OS and inflammation has been reported [9,10], probably affecting fetal growth and increasing the risk of having an SGA newborn. Excessive maternal fat mass or gestational weight gain has been directly involved in metabolic, inflammatory, and oxidative status alterations and may result in a higher risk of macrosomia or an LGA newborn [9]. However, in a retrospective cohort study, inadequate early weight gain was associated with an increased risk for SGA [11]. 

There may be insufficient protective mechanisms against increased OS in newborns from obese mothers and women with excessive weight gain. An imbalance between oxidative stress and total antioxidant capacity may lead to an accumulation of reactive oxygen species (ROS), which has been shown to decrease fetal growth via mTOR [7,8]. In a cross-sectional study, elevated malondialdehyde (MDA) levels, a marker for lipid oxidation, and decreased superoxide dismutase activity were observed in umbilical cord blood of SGA newborns compared to normal-weight newborns [8]. 

Other nutritional factors that have been associated with size at birth include vitamin D status and the use of multiple micronutrient supplementation (MMS). Moreover, in an individual patient data meta-analysis, MMS showed an overall lower risk of low birth weight and SGA [12]. Vitamin D deficiency appears to induce fetal intrauterine growth restriction. Experimental studies have shown that gestational vitamin D deficiency inhibits placenta development and function and is associated with increased inflammatory markers [13]. A recent meta-analysis has shown that maternal vitamin D supplementation (>600 IU/d) is associated with a lower risk of SGA and low birth weight [13,14]. Early prediction of SGA newborns brings an opportunity for implementing a more intensive follow-up with adequate nutrition and clinical strategies. Current efforts have been focused more on secondary prevention to reduce morbidity and mortality in SGA infants.

Artificial Neural Network models (ANN) have opened new perspectives in medicine forecasting since they do not hypothesize on data distribution (normality) and multicollinearity. They can predict complex combinations of categorical, scalar, and ordinal variables. ANN learns from the data (independent and dependent features called input and output variables) to simulate an outcome. Several studies predict fetal birth weight and growth by Machine learning models based on ultrasonographic data, maternal clinical characteristics, or serum biomarkers [15,16,17]. However, most of these predictive models have not considered the combination of maternal features, including obesity and early gestational weight gain or redox markers for predicting growth. This study aimed to develop an ANN model to simulate SGA newborns at birth based on early (first-trimester) maternal nutritional, metabolic, and oxidative status in pregnancy. The second outcome was to obtain the relative importance of each maternal factor. Then, several simulations were performed where the outcome was immediately displayed after varying the most critical maternal features attained by the ANN model to obtain different scenarios leading to SGA. 

## 2. Materials and Methods

### 2.1. Ethical Approval Statement

This study was approved by the Institutional Review Boards of the Instituto Nacional de Perinatología (INPer) (Protocol number: 2017-2-65), according to the Helsinki Declaration. Data were obtained from the OBESO (Epigenetic and Biochemical Origin of Overweight and Obesity) perinatal cohort (Approved protocol number: 3300-11402-01-575-17) and performed at the INPer in Mexico City. Participation was voluntary, and all women who agreed to participate signed the informed consent form. The patient names and personal information were eliminated to have an anonymized dataset.

### 2.2. Study Design

We first developed an early Artificial Neural Network (ANN) predictive model for SGA (Small for gestational age) or AGA (Adequate for gestational) neonates based on clinical, nutritional, biochemical data, and oxidative stress markers from the first trimester of pregnancy. The most critical maternal variables for estimating SGA or AGA will be obtained from this model. The secondary aim was to propose several predictive hypotheses for SGA based on manipulating the values of the most critical forecasting features obtained from the ANN model. The outcome is immediately displayed for each maternal feature value change to simulate several scenarios leading to SGA neonates.

OBESO is an institutional cohort of pregnant women and their children up to 2 years of age, which is aimed at studying the biochemical, clinical, lifestyle, and epigenetic determinants of obesity. Women were recruited at the Department of Maternal-Fetal Medicine in the first trimester of pregnancy. The sample was selected by convenience (January 2017–January 2019), according to inclusion criteria: healthy adult women, single pregnancy, without comorbidities (diabetes mellitus, renal or hepatic diseases, congenital malformations, autoimmune diseases, or uncontrolled thyroid disease), and not taking any medication that may affect endocrine metabolism (insulin, metformin, and/or corticosteroids). Women were eliminated from the analysis if they developed gestational diabetes, hypertension, preeclampsia during pregnancy, or incomplete data. Only 2 large for gestational age newborns were observed, so they were also eliminated from the analysis. All women received routine prenatal care at INPer. 

For this analysis, maternal data was obtained at the Nutrition Clinic in the first trimester of pregnancy (11–13.6 weeks of gestation). Gestational age at enrollment was calculated according to the fetal ultrasound performed during the first trimester. Women in the first visit reported pregestational weight. Current weight was measured to the nearest ±0.1 kg, with women wearing light clothing and no shoes, using a calibrated digital scale (BMB-800, TANITA, Tokyo, Japan). Height was measured to the nearest 0.1 cm using a digital stadiometer (model 264, SECA, Hamburg, Germany), with the head placed in the proper position, according to the Frankfort plane. The pregestational body mass index (pBMI) (weight (kg)/height (m^2^)) was computed, and the women were classified according to the WHO criteria [18] as follows: adequate weight (pBMI = 18.5 to 24.9), overweight (pBMI ≥ 25), or obese (pBMI ≥ 30). Gestational weight gain (GWG) during the first trimester was considered excessive if >2.0 kg, adequate if it was between 0.5 and 2.0 kg, and insufficient if <0.5 kg, according to the Institute of Medicine [19,20]. Body composition was measured by bioelectrical impedance (BIA) (Inbody 370, Inbody Co., Seoul, Korea) with women wearing light clothes, according to manufacturer’s instructions. Body fat data were computed from the equipment. Multivitamin supplementation (MVI) was prescribed by obstetricians or other health professionals involved in prenatal care; women were asked about supplement use. Infant anthropometric measurements were carried out at birth (first 24–48 h) by a trained nutritionist following the technique described by Lohman [21]. Infants were measured and weighed unclothed. Weight at birth was recorded using a pediatric scale (1582 Baby/Mommy Scale, Tanita, Tokyo, Japan). Recumbent length was measured by duplicate using an infantometer (SECA 207, SECA, Hamburg, Germany), and the average was computed. Weight-for-age was assessed using the WHO reference data for term infants (Anthro software v. 3.2.2, WHO, Geneva, Switzerland) [22] and the Intergrowth newborn data set for preterm infants [22,23]. SGA infants were classified when weight-for-age was <10 percentile. 

### 2.3. Samples, Biochemical Analysis, and Oxidative Stress Markers

A fasting maternal blood sample was collected in Vacutainer tubes (Becton-Dickinson, Franklin Lakes, NJ, USA) and centrifuged for 15 min at 1000× *g*. Serum samples were stored at −70 °C until the assays were performed.

Fasting serum triglycerides (Tgl), total cholesterol (Chol), Low-density lipoproteins (LDL-Chol), and high-density lipoproteins (HDL-Chol) concentrations were measured by enzymatic colorimetric methods using an automated analyzer (ISE Echo Lory 2000) and commercial kits (DiaSys Diagnostic Systems GmbH, Holzheim, Germany). The 25-hydroxyvitamin D (Vit D) concentration was performed by ELISA (chemiluminescence; Architect Abbott Diagnostics, Lake Forest, IL, USA). Malondialdehyde (MDA) is the end-product of lipoperoxidation and the most representative marker of oxidative lipid damage. MDA was quantified using 1-methyl-2-phenylindole, and absorbances at 586 nm were measured for each reaction [24]. Carbonylated proteins (CP) quantification in plasma, treatment with 2,4-dinitrophenyl hydrazine was used, which reacts with carbonyl groups to form stable hydrazones. These were then measured spectrophotometrically at 370 nm, according to the method described by Dalle and cols. [25] and expressed as pmol CP/mg protein. Total antioxidant capacity (TAC) as indicative of an antioxidant (AOX) defense system in plasma was evaluated according to a method based on cupric-reducing antioxidant capacity (CUPRAC), using copper (II) and neocuproine reagents; absorbance for each reaction was measured at 450 nm. The results were expressed as pmol Trolox equivalent/mg protein. Trolox is a water-soluble analog of vitamin E [26]. All markers evaluated in this study have high sensitivity and reproducibility and use validated methods that allow the assessment of oxidative stress. Vit D was considered adequate if >30 ng/mL and deficient if <20 ng/mL [27].

### 2.4. Artificial Neural Network Model Development and Validation

Data were obtained from the OBESO perinatal cohort and preprocessed with the participation of clinicians and researchers to select the most important variables. The database contained pregnant women in the first trimester of pregnancy, classified into two groups according to the neonatal weight for gestational age at birth: SGA or AGA (Table 1). The 14 features consisted of maternal clinical and biochemical variables and oxidative stress markers involved in overweight and obesity pregnancies. The maternal input variables were age (years), pBMI (kg/m^2^), first trimester variables: gestational weight gain (kg), fat mass (%), multivitamins (MVI; no/yes), triglycerides (Tgl; mg/dL), total cholesterol (Chol; mg/dL), HDL-Chol (mg/dL), LDL-Chol (mg/dL), Vit D (ng/mL), MDA (pmol MDA/mg dry weight), CP (nmol CP/mg protein), TAC (pmol of Trolox equivalent/mg of protein), and gestational age at birth (weeks). The outcome was SGA or AGA (codified as a binary category 0.5 or 1.0). 

The dataset was randomly divided into training (75%) and validation/test (15%) for the ANN model development. A 15% of the dataset (distinct from the training and validation datasets) was used to test the final model using performance measures described below as an independent dataset (Section 2.5). The artificial neural network (ANN) architecture comprises an input layer with the 14 maternal features (normalized), a hidden layer with activation functions, and an output layer: SGA or AGA prediction. Each input node is connected to the hidden layer nodes and the latter to the output node through Weights and biases (Wi, Wo, b1 and b2) coefficients. The model was developed as previously described by our group [28]. 

A feed-forward back-propagation neural network was used to train and validate/test the model. In the training stage, the back-propagation network training function (trainlm function) was used to change the Weights (Wi and Wo) and biases (b1 and b2) according to the Levenberg-Marquardt optimization algorithm (which was chosen because it is one of the fastest and first-choice algorithms as it does not need more memory). Training (learning) was performed according to training parameters in order to achieve a small Root Mean Square Error (RMSE) calculated from the real (experimental) and the network predicted values. RMSE was applied as the error function describing the performance of the network and was set to 10^−12^ (performance goal parameter).

The experimental dataset (75% of the entire database, defined as the training dataset) was used to train the ANN model. Only input variables in the database were normalized between the range of 0.1–0.9. Therefore, the entire dataset was scaled to a new value (*x*_i_) in that range, with the following equation:

$$x_{i}=0.8\;\left(\frac{x_{i}-x_{min}}{x_{max}-x_{min}}\right)+0.1$$

For the 10-fold cross-validation, the training database was split into 10 equal-sized parts, 9 parts were used for training and one part for validation. The training was evaluated by comparing the accuracy of the model with the validation set (15% of the entire database) and this was repeated 10 times, each time using a different part to estimate the performance, and each part was used as the validation set only one time. The validation set was used to adjust the architecture of the model and to minimize overfitting by comparing the accuracy of the model in each training using three different accuracy metrics, including the RMSE. This method allows the model to be trained on all available data (except for the test data). Each mother/neonate was contained in only one of the splits. Equations are shown in Appendix A.

Previous training and validation datasets from another database (distinct to the one used in this study) were used. The training dataset was used to train a network model in order to fix the trained learning rate and the number of iterations’ super-parameters. Then, the data of the validation dataset, was fed to this network with these super-parameters, and the deviation was obtained. According to the deviation, the learning rate and iterations’ super-parameters were compared, adjusted and the network was trained again until the best super-parameters were found. These super-parameters were then selected for this study. 1000 training epochs and a learning rate of 0.001 were found to result in a good model performance on the training and test sets.

For activation transfer functions, we did not select this super-parameter but applied several functions (hyperbolic tangent, (TANSIG), linear (PURELIN), or Log-Sigmoid (LOGSIG) in the hidden and output layers and compared the performance between the obtained models. The best performant model obtained used TANSIG and LOGSIG functions. As well, the number of nodes in the hidden layer was also fit starting with one, until the best performant model was obtained.

Three metrics measured the performance strength for both the cross-validation and validation/test: the Root Mean Square Error (RMSE, set to 10^−12^), the coefficients of the linear regression (R and R^2^) between the experimental and the simulated data, as well as the statistical slope and intercept test [29]. The latter compares the simulated and actual values (from the dataset) from the linear regression through a Student *t*-test analysis. The slope and intercept range must be close to 1 and zero, respectively, with a 99.8% confidence level. The program was run 30,000 times with 100 iterations by each neuron, beginning with one in the hidden layer. Matlab software (R2021a, Natick, MS, USA) and the Deep Learning Toolbox were used (we do not use the Matlab interface GUI-Matlab nnstart).

### 2.5. Predictive Performance of the Model

To further determine the predictive power of the trained/validated ANN model, the ability of the algorithm to discriminate between SGA (true positive) and AGA (true negative) newborns was evaluated on an independent test set (a different dataset, no part of which was used for learning and validation/test). The performance metrics were accuracy, F-1 score, positive and negative predictive values (PPV and NPV, respectively), and the Area Under the Receiver Operating Characteristics (AUROC).

### 2.6. The Relative Importance of the Maternal Variables in the Prediction

Maternal feature importance for predicting SGA or AGA was carried out with a sensitivity analysis calculated with the Garson Equation [30] and depicted as a percentage. This analysis allows maternal features to be ranked.

### 2.7. The Simulator of SGA

The simulator was created on an Excel spreadsheet. The equations of the learning ANN algorithm (Appendix A) were embedded together with the weights and biases of the final model. The 14 input maternal features were entered in the spreadsheet, and the effect on the SGA or AGA (output) was immediately calculated and displayed to show a simulation of distinct scenarios. The Excel spreadsheet is available upon request to the authors, but we plan to make this tool generally accessible for research purposes. 

### 2.8. Statistical Analysis

Quantitative variables in the first trimester were expressed as mean ± standard deviations (SD), while qualitative data were reported as percentages and numbers. Descriptive measures and frequencies were used to characterize the data. The Kolmogorov–Smirnov test was performed to compare numerical data distributions. Mean differences were analyzed with Student’s *t*-test (parametric data) and Mann-Whitney U test (non-parametric data). A Chi-square test was performed for categorical variables between groups. *p*-value < 0.05 was considered statistically significant. Statistical analyses were performed using SPSS v.25 (SPSS Inc., Chicago, IL, USA).

## 3. Results

### 3.1. Clinical Characteristics of the Population

From January 2017 to September 2019, 192 women from the OBESO cohort met the inclusion criteria for this study. Fifty women were eliminated because of developing gestational diabetes, preeclampsia, or gestational hypertension, and two newborns were classified as LGA. Of the remaining 140 women, 55% had complete first-trimester fat mass, oxidative stress, and lipid measurements. A final sample of 77 women and their newborns were studied: 18.2% (n = 14) of neonates were classified as SGA, while 81.8% (n = 63) were considered AGA. The mean maternal age of all women was 28 ± 5 years old. Before pregnancy, 57.1% (n = 44) of women were overweight or had obesity, the mean of GWG in the first trimester was 1.54 ± 3.19 kg, and the mean fat mass was 38.8 ± 7.12%. At some point during pregnancy, 1 woman reported using metformin and 5 women of steroids. No differences were observed in the SGA frequency between women using metformin/steroids and women who did not. Preterm birth (<37 weeks of gestation) was observed in 11.7% (n = 9) of women. When stratified by SGA or AGA outcome, no significant differences were observed in maternal anthropometric and biochemical characteristics (Table 1).

### 3.2. Development and Validation of SGA Predictive Model

**Figure 1:**
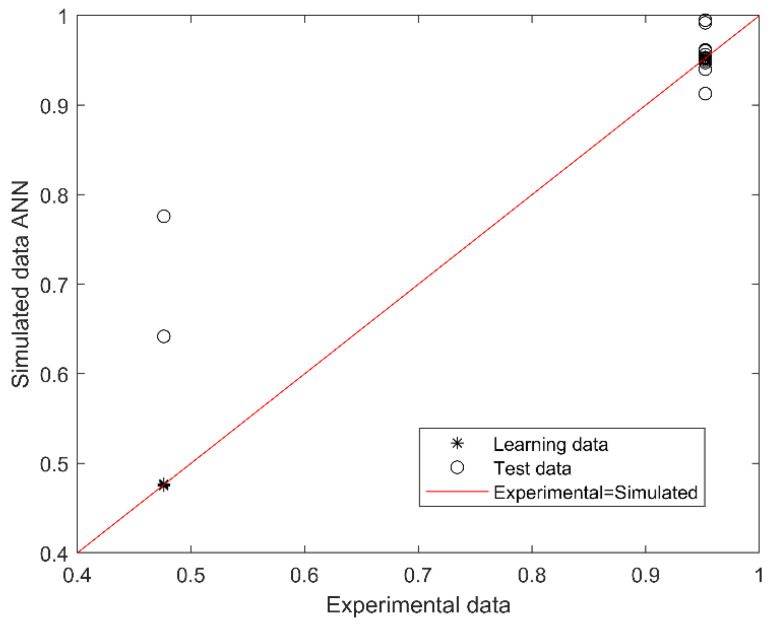
Scatter plot of experimental (actual) and simulated (predicted) data for SGA or AGA. The outcome (output of the model) is SGA or AGA (codified as 0.5 or 1.0). The red line indicates the linear regression model on scatter points, and the output is the best linear fit obtained by the ANN model. The range between 0.4 and 0.79 corresponds to SGA and between 0.8 and 1.0 to AGA prediction.

The best performing model for predicting SGA or AGA from 14 first trimester maternal nutritional, metabolic, and oxidative variables consisted of 2 neurons in the hidden layer (with Tansig and Logsig activation functions in the hidden and output layers, respectively; equations in Appendix A). The algorithm performance was verified based on the determination and correlation coefficients (R^2^ and R, respectively) between experimental (actual) and simulated data, analyzed through a linear regression (Figure 1). The overall model performance (all R, training, validation, and testing) was 0.969, and R^2^ was 0.938, indicating an accurate match between the observed (actual) and predicted data, with a 99.8% confidence in the slope and intercept Student’s *t*-test.

The outcome (output of the model) is SGA or AGA (codified as 0.5 or 1.0). The red line indicates the linear regression model on scatter points, and the output is the best linear fit obtained by the ANN model. The range between 0.4 and 0.79 corresponds to SGA and between 0.8 and 1.0 to AGA prediction.

### 3.3. Performance-Based on Confusion Matrix

Next, the model performance was evaluated on an independent dataset from training and testing (external validation). Overall, the model achieved an accuracy above 86%, F-1 score of 80%, PPV of 100%, NPV 80%, and an AUROC of 0.8.

### 3.4. The Relative Importance of Maternal Variables

**Figure 2:**
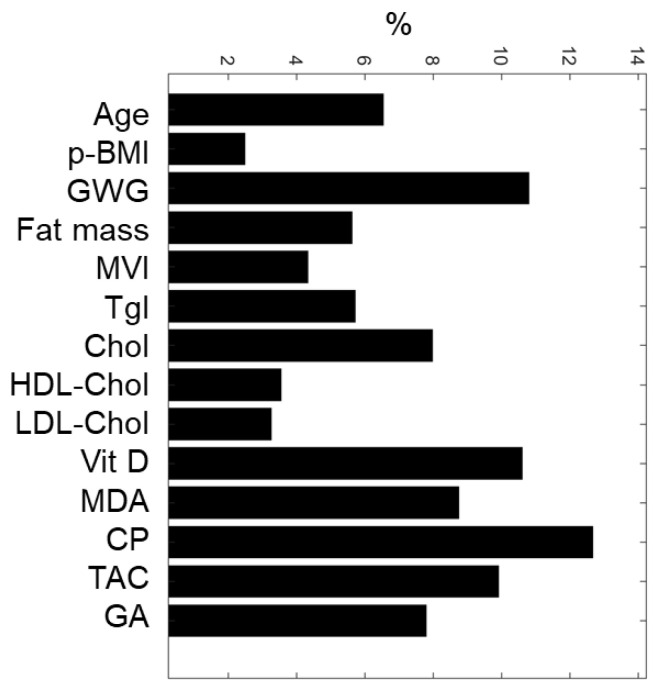
ANN maternal feature classification. Five maternal input variables with the highest relative importance for predicting SGA or AGA newborns were: CP, GWG, Vit D, TAC, and MDA. pBMI, pre-gestational BMI; GWG, gestational weight gain; MVI, multivitamins; Tgl, triglycerides; Chol, total cholesterol; HDL-Chol, HDL cholesterol; LDL-Chol, LDL cholesterol; Vitamin D, Vit D; MDA, malondialdehyde; CP, carbonylated proteins; TAC, total antioxidant capacity; GA, gestational age at birth.

Based on the weights associated with each maternal input variable, the sensitivity analysis of the ANN model allowed the classification of maternal nutritional, biochemical, and oxidative features (Figure 2). The top-five predictive maternal characteristics were protein oxidation (CP, 12.7%), gestational weight gain (GWG, 10.8%), vitamin D (Vit D, 10.6%), total antioxidant capacity (TAC, 9.9%), and lipid oxidation (MDA, 8.7%). In contrast, the least essential variables were pBMI (2.5%), total cholesterol (3.2%), and HDL-Cholesterol (3.5%). 

### 3.5. Simulator for SGA

**Figure 3:**
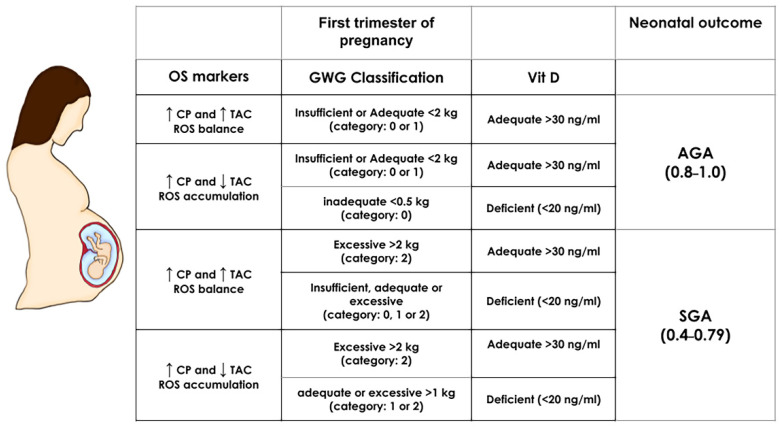
First-trimester SGA and AGA simulations in normal weight pregnancies. The prediction of neonatal outcome was calculated by manipulating the most important maternal predictors: oxidative stress biomarkers (OS markers CP and TAC), gestational weight gain (GWG), and vitamin D (Vit D) in normal weight pregnancies (the pBMI value was set at 24 kg/m^2^ and kept constant). The range for an SGA outcome is a numeric result between 0.4–0.79 and for AGA within 0.8–1.0 (based on the ANN algorithm Equation (A3) (Appendix A) and the scatter plot from Figure 1). Depicted are the values of maternal factors leading to an SGA or AGA result. GWG, gestational weight gain; TAC: Total antioxidant capacity; AGA: Appropriate for gestational age; SGA: Small for gestational age.

The equations of the ANN algorithm (Appendix A) and maternal inputs were embedded in an Excel spreadsheet to simulate distinct scenarios predicting SGA or AGA newborns. The most critical maternal features obtained by the model, i.e., first trimester oxidative stress biomarkers (CP, MDA, and TAC concentrations), GWG and Vit D status, were manipulated in normal weight pregnancies, and the effect on the outcome was immediately calculated (SGA result fell within the range of 0.45 and 0.79 while AGA between 0.8 and 1.0, from Figure 1) (Figure 3). Excessive GWG with adequate Vit D status in the first trimester predicts SGA even with a redox equilibrium balance. More importantly, Vit D deficiency independently of GWG and redox equilibrium balance or imbalance predicts an SGA newborn. We also obtained the simulations for obese pregnancies, but the predictions were the same as normal weight pregnancies (data not shown). This result is consistent with p-BMI as the less critical feature in the model.

## 4. Discussion

This study presents an integrative Artificial Neural Network (ANN) model for small for gestational age (SGA) prediction based on first trimester maternal nutritional, biochemical, and oxidative stress variables. The model obtained an excellent performance in the training/validation dataset using RMSE and regression coefficients criteria (>0.93), achieved an accuracy of 86%, and an area under the curve (AUROC) of 0.8 on an independent dataset.

Early estimation of abnormal fetal growth may allow the implementation of timely clinical and nutritional strategies with intensive follow-up during pregnancy to improve perinatal outcomes. Most published SGA or LGA (large for gestational age) forecasting models have been based on ultrasonographic, maternal characteristics, and placental biochemical data, using logistic regression and machine learning algorithms at different gestational weeks. Logistic regression models have reached AUROC values near 0.7 for SGA prediction with first trimester ultrasound data or the combination with placental biomarkers (including placental growth factor or soluble fms-like tyrosine kinase-1) [31,32]. Other studies showed that machine learning algorithms obtain more accurate predictions than logistic regression with higher AUROC values using only ultrasound and clinical characteristics from early pregnancy [15,16,17,33]. Fetal growth has also been estimated between 20–30 weeks of gestation by a machine learning algorithm based on ultrasound and fetal biometric data from INTERGROWTH-21st with promising results [34]. The work by Kuhle and cols. compared logistic regression and machine learning models for predicting SGA or LGA based only on maternal clinical characteristics at 26 weeks of gestation, reaching an AUROC ranging between 0.8–0.91 but did not find differences between algorithms [35]. The methods used in this study included logistic regression, Classification trees, Gradient boosting, and Elastic Net (EN). Recently, a study by Yamauchi and cols. constructed a model based on urinary metabolomics data using EN regression [36]. This is a hybrid model using multicollinearity and regularization as the optimization function and has been shown to work well for large datasets and highly correlated variables. Extreme gradient boosting (XGBoost) is an implementation of gradient boosted trees. It also performs well for large data and particularly for binary imbalanced classification, using trees as the weak learners. The method developed in this work does not take into account multicollinearity and performs very well for non-linear data as well as possible interactions between variables. A systematic review presented a summary of several machine learning models for the identification of adverse pregnancies [37].

The current study used an integrative approach to forecast SGA relying on pBMI and first-trimester fat mass, weight gain, vitamin D, and redox status (lipid and protein oxidation together with antioxidant capacity) as inputs for the algorithm. No models in the literature have used such a combination of maternal predictors that include nutritional, metabolic, and dysregulation of redox balance, with intersecting roles in pregnancy. We believe that the approach presented in this work, although with its limitations, could improve clinical follow-up by early estimation of a frequent and relevant adverse perinatal outcome, having an SGA newborn. One study has integrated anthropometric variables such as gestational weight gain (GWG), pBMI, and age to predict SGA or LGA combined with neonatal lean and fat to measure infant adiposity [38]. The use of ANN models in this study allowed us to evaluate the effect of many variables together with a high level of precision.

Excessive gestational weight gain has been mainly associated with having an LGA newborn [39]. However, gestational weight gain (with smoking and a previous low birth weight infant) were predictors of SGA at 26 weeks gestational age by machine learning and logistic regression methods [35]. At present, there is controversy about whether pre-gestational BMI or GWG have more impact on fetal growth [40]. Our results showed that first-trimester GWG, maternal protein and lipid oxidation markers, and vitamin D concentrations were stronger predictors of SGA when compared to p-BMI, which was the less important feature. This evidence is interesting because most studies of obesity and fetal growth have been carried out with p-BMI, without considering other important factors, including fat mass. Even though BMI is a valid adiposity index, it is not a measure of fat mass. In our study, first-trimester maternal fat mass was not a strong predictor of SGA, but it was more relevant than p-BMI.

Using our models, excessive GWG was a strong predictor of SGA deliveries, independently from the balance or imbalance of reactive oxygen species/antioxidant (ROS/AOX). In the context of oxidative stress, early excessive adiposity (weight/fat mass gain) may be affecting the nutrient transfer and sensing pathways in early pregnancy, resulting in impaired fetal growth. The pathogenesis of obesity is complex and includes metabolic and hormonal dysregulation, low-grade chronic inflammation, and endoplasmic reticulum stress, among other closely interconnected processes [13,14,41]. More studies considering pBMI in addition to fat mass and early weight gain are needed to understand the possible effect of rapid weight gain on fetal growth and size at birth. 

Vitamin D deficiency was a strong determinant of SGA, independently from GWG. Vitamin D is known to play a vasoprotective effect by reducing oxidative stress-induced endothelial dysfunction and regulating the bioavailability of nitric oxide [42]. Experimental evidence has demonstrated placental development inhibition and impaired function when vitamin D deficiency is induced [14,41]. Even though the precise mechanism by which vitamin D deficiency-induced placental insufficiency and fetal IUGR is not known, increased inflammation appears to be a relevant factor [42,43]. In human studies, there is controversy about the association between vitamin D deficiency and size at birth [44]. A recent meta-analysis of observational studies has shown a higher risk of SGA with vitamin D concentrations < 20 ng/mL and lower birth weight, length, and head circumference in newborns of women with lower vitamin D concentrations (<12 ng/mL) [45]. Consistent with our study, other meta-analyses have documented the association between vitamin D deficiency and a higher risk of SGA [46,47,48]. In addition, vitamin D supplementation during pregnancy (600 IU/d vs. placebo or not receiving vitamin D) reduces the risk of low birth weight and SGA [14]. This is very relevant considering the high prevalence of maternal vitamin D deficiency worldwide. In a recent cohort study in Mexico City, we reported 37% of vitamin D deficiency in the first trimester of pregnancy, and 20% in the third trimester, even though 76% of mothers were receiving some type of vitamin D supplementation [27]. 

In addition to GWG and vitamin D, early oxidative stress markers were strong predictors of SGA. In intrauterine growth restriction (IUGR) complicated pregnancies, an increase in oxidative markers (MDA, isoprostanes, protein carbonyls) in the placenta, maternal, and cord plasma has been observed [38,49,50,51]. In our study, the stronger oxidative stress predictors of SGA were CP, MDA, and TAC. Some of the mechanisms that have been studied and that may associate oxidative stress with fetal growth impairment (IUGR or SGA) include defective arterial modeling, reduced nutrient and oxygen supply, increased ROS, MDA, and isoprostanes, as well as upregulation of antioxidant enzymes (superoxide dismutase and glutathione peroxidase) and antioxidant depletion (vitamin E and glutathione) [52]. Many of these factors may be associated with excessive fat mass. 

We assessed important nutrition and metabolic factors that may predict alterations in fetal growth. However, there are many other lifestyle, clinical and sociodemographic variables that have been associated with low birthweight or SGA. Macronutrient imbalances, dietary patterns, physical activity, stress, sleep patterns, inflammation, insulin and adipokine levels, placental status, and oxygen flow, among others, may modify hormonal and metabolic processes that regulate and affect nutrient transfer and fetal growth [7].

Primary prevention of SGA newborns is a challenge and represents a global goal. Very few interventions have proven successful in reducing SGA and/or IUGR: multiple micronutrient supplementation [12], balanced energy, and protein supplementation [53] in undernourished mothers, vitamin D supplementation at specific doses [14], among few others. Most current interventions are aimed to reduce morbidity and mortality in infants already born SGA.

The implementation of timely intervention strategies may reduce the prevalence of SGA. It has been estimated that a reduction of the prevalence of SGA from 19.3% to 10.0% in low to middle-income countries could reduce neonatal deaths by 9.2% [4]. It is important to note that the prevalence of SGA may differ according to the method used to ascertain gestational age (ultrasound, last menstrual date), the time when the measurement was performed (at birth, 24–48 h post-birth), and the reference growth curved selected for evaluating weight/age.

Achieving an optimal nutrition status by offering intensive counseling about healthy eating and prescribing individualized nutrient supplementation schemes is critical to prevent multiple perinatal complications. Good nutrition is essential to promote and maintain an adequate gestational weight gain, optimal antioxidant capacity, and good vitamin D status, three factors that, according to our results, were strong predictors of SGA.

### 4.1. Strengths and Limitations of the Study

This study has some limitations that need to be addressed. First, weighing women before pregnancy was impossible, so self-reported p-BMI was used; this may introduce bias in their weight classification and GWG estimation. Second, the dyad mother-newborn sample size is relatively small; however, the model accurately estimated AGA or SGA with an R > 0.93. Although the external validation was carried out with an independent dataset, never used for the model’s training and validation/test, it will be necessary to have additional external datasets from other health care centers for further training and validation of this model. Third, since this study was conducted in a tertiary health center, the prediction may not apply to all pregnant women, making it difficult to generalize the results and not entirely represent the general obstetric population. Despite this, we considered an advantage of this study to include a population of pregnant women with different weight statuses. 

Strengths of this study include that the dataset is derived from a longitudinal cohort that allowed us to include first trimester data and the use of redox biomarkers as fetal growth predictors. It is important to mention that the latter may also be a possible weakness because that some of these measurements cannot be performed in low-resource settings. However, the study design was not intended to be used in such environments but rather as a more personalized prediction tool for an SGA outcome considering the complex metabolic alterations and dysregulation of redox balance.

### 4.2. Challenge and Future Perspectives

Lipid and protein oxidation markers (MDA and PC, respectively) and TAC are biomarkers that provide evidence of oxidative damage. However, this evaluation could be supplemented by other redox markers, such as quantifying isoprostanes and glutathione-dependent enzyme activity. To further complete the prediction and monitoring of fetal growth, second and third-trimester maternal features will be incorporated into a new model with the first-trimester features from this study to personalize the outcome of an SGA newborn further. In this sense, the pre-processing of signals in time-series data would be highly enriched using filters, such as the Savitzky-Golay algorithm and the 1-D wavelet decomposition, that eliminate the possible outliers and smooth the signal, which leads to more accurate predictions. Such filters have been shown to reduce noise in data [54] and will be addressed in future work to remove noise in metabolomic data and ultrasound images; maternal features that could be incorporated into predictive models.

## 5. Conclusions

Compared to models based on ultrasound and anthropometric measurements, the novel combination of maternal predictors, including p-BMI, fat mass, weight gain, biochemical, and redox status (balance or imbalance of reactive oxygen species/antioxidants) in first-trimester, provided accurate and personalized forecasts of SGA neonates.

Strengths of the model:

The database was collected from a population that included pregnancies with or without obesity, which has been associated with an increased risk of adverse fetal growth.

The ANN model used three different performance metrics compared to other machine learning models in the training/validation datasets and additional predictive power measurement (AUROC) on an independent dataset.

The implementation of a simulator on an Excel spreadsheet allows forecasts by the model.

Ranking of the maternal predictive features showed that protein and lipid oxidative stress markers, total antioxidant capacity, gestational weight gain, and vitamin D were key for SGA prediction.

## Figures and Tables

**Table 1:** First trimester maternal clinical and biochemical data and gestational age according to newborn weight for age.

Variables	All Women (n = 77) Mean ± SD n (%)	SGA (n = 14) Mean ± SD n (%)	AGA (n = 63) Mean ± SD n (%)
Age (years)	28 ± 5	29 ± 4	28 ± 5
Parity:			
Nulliparous	35 (45.5)	7 (50)	28 (44.4)
Multiparous	42 (54.5)	7 (50)	35 (55.6)
Socioeconomic Status:			
Low/lower-middle-income	49 (63.9)	11 (78.6)	28 (60.3)
Upper middle-/high-income	28 (36.4)	3 (21.4)	25 (39.7)
p-BMI (kg/m^2^)	26.9 ± 5.5	28.2 ± 8.0	26.6 ± 4.9
p-BMI group:			
Normal	33 (42.9)	5 (35.7)	28 (44.4)
Overweight/obesity	44 (57.1)	9 (64.3)	35 (55.6)
GWG (kg)	1.5 ± 3.2	2 ± 3.1	1.4 ± 3.2
Fat mass (%)	38.8 ± 7.1	39.7 ± 8.9	38.6 ± 6.8
MVI supplementation:			
Yes	28 (36.4)	4 (28.6)	24 (38.1)
No	49 (63.6)	10 (71.4)	39 (61.9)
Medication:			
Yes	5 (6.5)	1 (7.1)	4 (6.3)
No	72 (93.5)	13 (92.9)	59 (93.7)
Glucose (mg/dL)	80.8 ± 9.6	80 ± 11.4	81 ± 9.3
Triglycerides (mg/dL)	136 ± 46.4	157 ± 63.4	132 ± 41.1
Total Cholesterol (mg/dL)	187 ± 38.5	201 ± 32.6	184 ± 39.3
HDL-Cholesterol (mg/dL)	60.5 ± 12.4	59.7 ± 11.1	60.7 ± 12.8
LDL-Cholesterol (mg/dL)	92.1 ± 25.6	89.9 ± 27.9	92.7 ± 25.3
HbA1c (%)	5.3 ± 0.4	5.2 ± 0.5	5.3 ± 0.4
25-OH-D (ng/mL)	21.6 ± 6.8	19.9 ± 3.4	22 ± 7.2
MDA (pmol MDA/mg dry weight)	170 ± 174	153 ± 180	173 ± 173
CP (pmol CP/mg protein)	5397 ± 2617	5710 ± 2388	5327 ± 2679
TAC (pmol of Trolox equivalent/mg protein)	81.1 ± 28.4	78 ± 30.7	81.8 ± 28
Term birth:			
Yes	68 (88.3)	12 (85.7)	56 (88.9)
No	9 (11.7)	2 (14.3)	7 (11.1)
Newborn sex:			
Female	39 (50.6)	5 (35.7)	34 (54)
Male	38 (49.4)	9 (64.3)	29 (46)

p-BMI: Pregestational Body Mass Index; GWG: Gestational weight gain; MVI: Multivitamin; HDL: High-density lipoprotein cholesterol; LDL: Low-density lipoprotein cholesterol; HbA1C; Hemoglobin A1c; 25-OH-D: 25-hydroxyvitamin D; MDA: Malondialdehyde; CP: Carbonylated proteins. TAC. Values represent mean ± SD.

## Data Availability

Data con obtained from the authors. The data are not publicly available due to ethical restrictions.

## Appendix A

In the hidden and output layer, each neuron (*n*) has weights (*Wi* and *Wo)* and biases (*b*1 and *b*2), in the hidden and outputs layers, respectively, (see Equations (A1) and (A2)):

*n*_1_ = *WiIn*_1_ + *WiIn*_2_ + ……. + *WiIn*_k_ + *b*1 (A1)

where *In* are the Input variables.

The value of each neuron is the argument of the transfer functions (*f* and *g*):

*SGA or AGA* (*Output*) = *g*(*Wo* × *f* (*Wi* × *In* + *b*1) + *b*2) (A2)

where *f* is a hyperbolic tangent (TANSIG) activation function in the hidden layer and *g* is a Log-sigmoid (LOGSIG) transfer function in the output layer. These two transfer functions obtained the best performant model. As a result of Equation (A2) with these functions:

Equation (A3) gives *SGA* or *AGA* with TANSIG-LOGSIG with weights and biases:

(A3) $$SGA\;or\;AGA\;\left(Output\right)=\frac{1}{\left(1+EXP\left(-n_{output\;layer}\right)\right)}$$

where *output layer* is:

$$output\;layer=\sum_{s}\left\{Wo_{\left(l,s\right)}\cdot \left[\frac{2}{1+e^{-2\cdot \left(\sum_{k}Wi_{\left(s,k\right)}\cdot In_{k}+b1_{\left(s,1\right)}\right)}}-1\right]\right\}+b2_{\left(l,1\right)}$$

where:

$$\underset{k}{\sum }Wi_{\left(s,k\right)}\cdot In_{k}+b1_{\left(s,1\right)}=X1+X2$$

and *X*1 and *X*2 are defined as:

$$X1=\left(\begin{array}{l} W_{i\left(1,1\right)}I_{1}+W_{i\left(1,2\right)}I_{2}+W_{i\left(1,3\right)}I_{3}+W_{i\left(1,4\right)}I_{4}+W_{i\left(1,5\right)}I_{5}+W_{i\left(1,6\right)}I_{6}+W_{i\left(1,7\right)}I_{7}\ldots \\ +W_{i\left(1,8\right)}I_{8}+W_{i\left(1,9\right)}I_{9}+W_{i\left(1,10\right)}I_{10}+W_{i\left(1,11\right)}I_{11}+W_{i\left(1,12\right)}I_{12}++W_{i\left(1,13\right)}I_{13}+W_{i\left(1,14\right)}I_{14}+b1_{\left(1\right)} \end{array}\right)$$

$$X2=\left(\begin{array}{l} W_{i\left(1,1\right)}I_{1}+W_{i\left(1,2\right)}I_{2}+W_{i\left(1,3\right)}I_{3}+W_{i\left(1,4\right)}I_{4}+W_{i\left(1,5\right)}I_{5}+W_{i\left(1,6\right)}I_{6}+W_{i\left(1,7\right)}I_{7}\ldots \\ +W_{i\left(1,8\right)}I_{8}+W_{i\left(1,9\right)}I_{9}+W_{i\left(1,10\right)}I_{10}+W_{i\left(1,11\right)}I_{11}+W_{i\left(1,12\right)}I_{12}++W_{i\left(1,13\right)}I_{13}+W_{i\left(1,14\right)}I_{14}+b1_{\left(1\right)} \end{array}\right)$$

and input variables (*I*) were:

*I*_1_ = *age*; *I*_2_ = *pBMI*; *I*_3_ = *GWG*; *I*_4_ = *fat mass*; *I*_5_ = *MVI*; *I*_6_ = *triglycerides*; *I*_7_ = *Chol*;

*I*_8_ = *HDL-Chol*; *I*_9_ = *LDL-Chol*; *I*_10_ = *Vit D*; *I*_11_ = *MDA*; *I*_12_ = *CP*; *I*_13_ = *TAC*; *I*_14_ = *GA*
